# Exploring the role of emotional intelligence on disorder eating psychopathology

**DOI:** 10.1007/s40519-018-0629-4

**Published:** 2018-12-19

**Authors:** Una Foye, D. E. Hazlett, Pauline Irving

**Affiliations:** 1 Centre of Psychiatry, Queen Mary University of London, London, UK; 2 Centre for Mental Health Research, School of Health Sciences, City, University of London, Northampton Square, London, EC1V 0HB UK; 3 Centre of Higher Education Research and Practice, Ulster University, Jordanstown Campus, Newtownabbey, Co. Antrim UK; 4 Faculty of Social Sciences, Ulster University, Jordanstown Campus, Newtownabbey, Co. Antrim UK

**Keywords:** Emotion, Emotional Intelligence, Psychopathology

## Abstract

**Purpose:**

This study aims to explore the role of Emotional Intelligence (EI) and specific facets that may underpin the aetiology of disordered eating attitudes and behaviours, as a means to understand what aspects of these deficits to target within treatments.

**Methods:**

Participants were recruited from the UK and Ireland. Among the sample of 355 participants, 84% were women and 16% were men. Regarding age, 59% were between 18 and 29, 30% were between 30 and 49, and 11% were 50 or older. Using a cross-sectional design, participants completed the Schutte Self-Report Emotional Intelligence Test to measure levels of trait EI and The Eating Attitudes Test (EAT-26) as a measure of eating disorder risk and the presence of disordered eating attitudes.

**Results:**

EAT-26 scores were negatively correlated with total EI scores and with the following EI subscales: appraisal of own emotions, regulation of emotions, utilisation of emotions, and optimism. Also, compared to those without an eating disorder history, participants who reported having had an eating disorder had significantly lower total EI scores and lower scores on four EI subscales: appraisal of others emotions, appraisal of own emotions, regulation of emotions, and optimism.

**Conclusions:**

Considering these findings, EI (especially appraisal of own emotions, regulation of emotions, and optimism) may need to be addressed by interventions and treatments for eating disorders.

**Level of evidence:**

Level V, descriptive cross-sectional study.

## Introduction

Salovey and Mayer [1] described emotional intelligence (EI) as: “…the ability to perceive emotions, to access and generate emotions so as to assist thought, to understand emotions and emotional knowledge, and to reflectively regulate emotions so as to promote emotional and intellectual growth.” (p. 5).

Studies have shown that psychological health is related to an individual’s emotional competencies and functioning [2]. Studies show that higher levels of EI are linked to both psychological and physical health, including findings that have found positive effects on psychological well-being and physical health, e.g. immune systems functioning [3]. As individuals with high EI have increased emotional resources, they are better equipped to deal with difficult life events; the emotionally intelligent individual is able to perceive, interpret and understand emotional and situational cues better than those with low EI and, thus, are more prepared and able to manage and effectively regulate emotional states and engage with adaptive problem solving [4]. Conversely, those who are low in EI are unable to effectively understand, regulate and control their emotions, therefore, will be less effective in coping suggesting that they are more likely to engage in maladaptive coping or emotional regulation strategies [5]. This has been shown across studies in which participants with low levels of EI have been found to be more likely to engage in maladaptive health behaviours as an attempt to seek alternative methods of mood control and emotional expression such as smoking [6], alcohol use [7] and self-harm behaviours [8].

### Emotional intelligence and eating disorders

The role of emotions in eating disorders is not new. Within her seminal works, Hilde Bruch was among the first to address the role of emotional disturbances as smokescreen to the emotional difficulties faced by those suffering from an eating disorder [9]. Explorations of the eating disorder experience have shown the important role of the eating disorder as having a functional purpose related to emotional regulation and coping [10–14]. These findings have been further expanded and focused to show deficits within this population in emotional processing, regulation and awareness [15–23].

In addition, literature has found evidence for deficits within areas of emotional processing including understanding one’s own emotions and facial processing in relation to appraising others emotions [24–26]. Somatic awareness and arousal have also been reported as dampened within these groups [27]. Based on this understanding that emotional deficits are strongly associated with disordered eating, a range of emotion-focused treatments have been explored with evidence supporting the acceptability and feasibility this approach for anorexia [28].

These findings correlate with the well-established relationship that exists within the literature between eating disorders and the psychological construct of Alexithymia, defined as a construct meaning literally “…without words for emotions” which described a severe deficit in an individual’s representation of emotions symbolically and the understanding, processing and describing of emotions [29, 30]. Empirical findings have formulated this construct to include features such as difficulty in describing and identifying feelings, difficulty in distinguishing between feelings and bodily sensations of emotional arousal, constricted imaginative processes, e.g. paucity of fantasies and externally oriented cognitive style [31]. The highest rates of alexithymia have been found in eating disorder patients with rates of alexithymia within anorexia nervosa patients between 23–77%, and 40–63% in bulimia nervosa patients; compared to rates of 0–28% in non-clinical samples [32–34]. With eating disorders being highly related to alexithymia, this may provide insight into the low success rates within treatments and psychotherapy as clients with high alexithymia scores have been found to be less able to respond to such interventions due to low insight and inability of emotional expression [35, 36].

While alexithymia research has found significant evidence for its role as a maintenance factor within eating disorder models, these findings express severe dysfunction and clinical levels of eating disorders, thus continuing the trend in eating disorder literature to view these behaviours as polarised. The introduction of a spectrum of emotional functioning, and dysfunction, across the range of emotional aspects described above can be found within the theoretical concept of EI.

The core elements of EI can be closely related to the emotional deficits that have been found within eating disorder populations. This suggests that individuals exhibiting disordered eating attitudes and behaviours, who experience such deficits in their emotional resources, have low levels of EI. Studies have provided evidence towards this application with results showing that those with high disordered eating have significantly lower EI than general populations [37–45]. The findings across these papers show that the relationship between EI and disordered eating spans across adults and adolescents, as well as occurring within sporting populations. These findings support theories linking negative affect and emotion dysregulation with the development of eating disorders, highlighting potential benefits of utilising EI for the identification of at-risk populations or as a potential screening measure for the issue. Furthermore, results highlight the role of emotion in disordered eating attitudes, which is an important finding in terms of the prevention and management of disordered eating.

While these studies provide strong evidence of the relationship between EI and eating disorders, the nature of what elements of EI are salient remains unclear. While there is theoretical recognition that emotional aspects of eating disorders encompass the interpersonal and intrapersonal [16], the majority of the literature places a focus on recognition and coping [12–15, 19, 21, 46] and to a lesser extent aspects of recognition and awareness [20, 23, 24]. Absent from this literature are facets of EI optimism and utilisation of emotion. Utilisation of emotions has been defined as the components of flexible planning, creative thinking, redirected attention and motivation [47] and optimism includes expectations of control over one’s own positive results in the future as well as a component of personal effectiveness [48]. To apply recommendations of literature surrounding the entire EI construct as an intervention for screening, treatment and prevention [37–45] requires further investigation of these facets that have been missed within the literature to ensure that programmes developed are able to utilise the correct facets and target the aspects of EI that are relevant.

### Aim

The application of emotional dysfunction theories may provide insight into why treatment for eating disorders remains blighted by the high levels of ambivalence, treatment dropouts and relapse rates that are observed within this patient population [49]. As a result, these disorders observe high rates of chronicity and become incredibly difficult to treat as they are so deeply engrained [50]. The aim of this study is to assess the link between trait EI and disordered eating attitudes to understand the nature of the relationship reported in the literature.

We hypothesise that disordered eating attitudes will be associated with lower EI scores; however, hypothesis that this relationship will be driven by core facets of the EI definition around management and regulation of emotions with facets related to the ability to access and express emotions remains less relevant. We believe understanding the nature of the relationship between EI and eating disorders will allow treatments to focus more effectively on the core facets of the disorder rather than more broadly with the vast area of emotions.

## Methodology

The findings reported within this paper are part of a larger UK-based study which explored the variables and barriers impacting on the onset and maintenance of eating disorders. The study used a sequential, mixed methods design made up of two phases. Following this research design, the current paper will explore the data emerging related to emotional functioning.

### Participants

Participants were recruited in the UK and Ireland from two local universities and a local further education institution, three community groups focused on the area of eating disorders, and five general health organisations. To ensure anonymity data collected, we did not note where participants were recruited from. Groups were encouraged to share the study link for additional snowball sampling [51]. Exclusion criteria included participants under 16 years of age, individuals in treatment, or those living outside of the UK or Ireland. From these sampling procedures, 435 agree to participate, and of them, 355 completed all survey measures that were included in the data analyses. Among this final sample of 355, 84% were women and 16% were men. Regarding age, 59% were between 18 and 29, 30% were between 30 and 49, and 11% were 50 or older. From this final sample, 23% identified themselves as having had an eating disorder, with 29 reporting more than one of these disorders at different points in time (41 with anorexia, 21 with bulimia, 28 with binge eating disorder, and 29 with eating disorder not otherwise specified). Of these 83 individuals who reported having had an eating disorder, only 42 reported having received treatment, 20 received treatment for anorexia (*n* = 20), five for EDNOS, three for bulimia, three for binge eating disorder and 11 for complex, multiple or atypical presentations. Females accounted for the majority of those having received treatment (*n* = 41) with only one male reporting having received treatment for their eating disorder. Due to the unequal distribution between eating disorder experiences, non-parametric testing was used to carry out the data analysis related to this variable.

### Questionnaires and procedure

Institutional ethical approval was granted by Ulster University’s Research Ethics Committee prior to the study commencing. The questionnaires below, along with a demographic survey, were placed online for completion by participants with a web link to allow the study to be distributed widely. When participants accessed the link to the survey, they were first required to give informed consent.

#### Schutte Self-Report Emotional Intelligence Test

EI was measured using the Schutte Self Report EI Test [47]. This measure was selected as this measure of EI is one of the most widely used scales and can be used by a wide variety of participant groups. The SSEIT is a 33-item measure that uses a 5-point likert scale to measure levels of trait EI. The measure represents all portions of the Salovey and Mayer model of EI [1], with 13 items representing the appraisal and expression of emotion category, e.g. I know when to speak about my personal problems to others, 10 items representing the regulation of emotion, e.g. I have control over my emotions and 10 items representing the utilisation of emotion, e.g. I motivate myself by imagining a good outcome to tasks I take on. This measure has been widely used within the literature testing Emotional Intelligence levels. Reports show the measure to have an internal consistency of 0.90, with a 2-week test–retest reliability of 0.78 from the same sample [47] with further validation studies carried out by Austin et al. [52]. The Cronbach alpha coefficient was 0.897. Scored ranged between 67 and 162 with a mean of 119.738 (SD 14.251, trimmed mean 120.050).

The EI scale was also recorded using the subscales, according to the analysis by Lane et al. [53] to provide a scoring for six facets of EI; Appraisal of Others Emotions consisting of seven items and an internal consistency of 0.79, Appraisal of Own Emotions consisting of five items and an internal consistency of 0.77, Regulation consisting of five items and an internal consistency of 0.6, Social Skills consisting of five items and an internal consistency of 0.62, Utilisation of Emotions consisting of seven items and an internal consistency of 0.67, and Optimism consisting of four items and an internal consistency of 0.66.

#### Eating Attitudes Test (EAT-26)

The Eating Attitudes Test (EAT-26) [54] was selected as a measure of eating disorder risk and presence of disordered eating attitudes as this is the most widely utilised means of disordered eating. The EAT-26 is a non-clinical self-report 26 item measure of individuals disordered eating attitudes. This measure has replaced the 40 item Eating Attitudes Test as one of the most widely used measures of disordered eating, including questions related to dieting and pre-occupation with being thinner; “I am terrified about being overweight”, bulimia and food pre-occupation; “I have the impulse to vomit after meals”, and oral control; “I avoid eating when I am hungry”. Questions are scored on a 4 point likert scale ranging from Always scored as 3, Usually scored as 2, Often scored as 1 and Never as 0; one item is reverse scored with Never scoring 3 and Always scoring 0. Scores at or higher than 20 indicate high levels of concern about dieting and possible disordered eating in non-clinical samples. Research has shown that the EAT-26 has an accuracy rate of at least 90% when used to differentially diagnose those with and without eating disorders and that mean EAT-26 scores differed among eating-disordered, symptomatic, and asymptomatic participants [55]. The measure has demonstrated internal consistency of between 0.79 and 0.94 [54, 55].

### Data analysis

Data collected from the study were analysed using SPSS 21.0. Preliminary statistical tests to identify linearity, outliers and distribution were carried out to test the validity of these data. Pearson correlation analyses were conducted to explore the relationships between EAT-26 and all EI measures, first for all participants combined and then separately for participants with and without an eating disorder history. To further understand these relationships, a series of Mann–Whitney *U* tests were conducted to examine differences in the EI measures between those with and without an eating disorder history.

## Results

Preliminary analyses revealed that those with a history of having an eating disorder had significantly higher EAT-26 scores (*M* = 35.46) than those with no history of an eating disorder (*M* = 17.13), *U* = 3431.00, *Z* = − 9.22, *p* < 0.001. There was no significant difference in EAT-26 scores between those who had treatment for their eating disorder and those who had no treatment.

Table 1 shows the results of the Pearson correlation analyses that tested the relationships between EAT-26 scores and both the EI total score and EI subscale scores. For all participants combined, whereas there was a significant negative correlation between EAT-26 scores and total EI score, this relationship remained significant for only four facets of the EI construct: appraisal of own emotions, emotional regulation, emotional utilisation, and optimism. As indicated previously, these analyses were conducted again but separately for the different groups. For those who reported having had an eating disorder, these same relationships remained significant. For those with no history of an eating disorder, the relationships with emotional regulation and emotional utilisation were no longer significant.

**Table 1:** Correlations between EAT-26 scores and EI subscale scores

Scale	Total sample (*n* = 355)	Group 1: history of an ED (*n* = 83)	Group 2: no ED (*n* = 272)
Overall EI	*r* = − 0.221, *p* < 0.001	*r* = − 0.371, *p* < 0.001*	*r* = − 0.118, *p* = 0.064
Appraisal of Own Emotions	*r* = − 0.332, *p* < 0.001*	*r* = − 0.363, *p* = 0.09*	*r* = − 0.280, *p* < 0.001*
Emotional Regulation	*r* = − 0.335, < 0.001*	*r* = − 0.477, *p* < 0.001*	*r* = 0.085, *p* = 0.2840.05
Emotional Utilisation	*r* = − 0.272, *p* = 0.005*	*r* = − 0.346, *p* = 0.004*	*r* = 0.030, *p* = 0.656
Optimism	*r* = − 0.059, *p* < 0.001*	*r* = − 0.379, *p* = 0.001*	*r* = − 0.131, *p* = 0.033*
Appraisal of Others Emotions	*r* = 0.059, *p* = 0.291	*r* = 0.158, *p* = 0.171	*r* = − 0.061, *p* = 0.338
Social Skills	*r* = − 0.056, *p* = 0.313	*r* = − 0.092, *p* = 0.426	*r* = 0.008, *p* = 0.898

*Indicate a significant result

Results of the Mann–Whitney *U* analyses that tested whether the EI total and subscale scores differed between groups. Compared to those without an eating disorder history, participants who reported having had an eating disorder had significantly lower total EI scores and lower scores on four facets of EI: appraisal of others emotions, appraisal of own emotions, emotional regulation, and optimism.

## Discussion

Currently, there is no clear aetiological model for eating disorders, with a wealth of causal models suggested to explain their onset [56]. With a considerable range of biological, psychological and social theories evidenced within the wide range of literature, the results from this study provide insights into the aspects of emotional dysfunction that may influence the psychopathology of disordered eating.

The results of this study found that individuals with high EI had significantly lower disordered eating attitudes, while those with low EI had significantly higher levels of disordered eating attitudes. This supports previous research in which EI is strongly related to disordered eating attitudes and behaviours with low EI scores predictive of higher levels of disordered eating [37]. These finding suggest that EI skills may provide protection against the development of disordered eating attitudes while their absence create a vulnerability. These findings support previous literature that has found this relationship between disordered eating and EI [44, 45, 57–59]. These results add to our understanding of the nature of this relationship and leads to questions regarding how low EI may contribute to the development of eating disorders.

Overall, there is a depth of research demonstrating a salient relationship between EI and disordered eating attitudes as well as the emerging evidence of the specific facets of EI that may contribute to this relationship. This may present an opportunity to tackle these illnesses from a secondary intervention perspective. Secondary prevention can produce targeted programs in which EI skills such as problem-solving can be built alongside emotional psychoeducation as a means to build coping mechanisms and resilience. By changing perspective from interventions to remove the triggers and difficulties that create risk for individuals, the evidence that high EI may protect against onset of disordered eating attitudes provides the opportunity to build the individual to cope with and manage the risk.

**Fig. 1:**
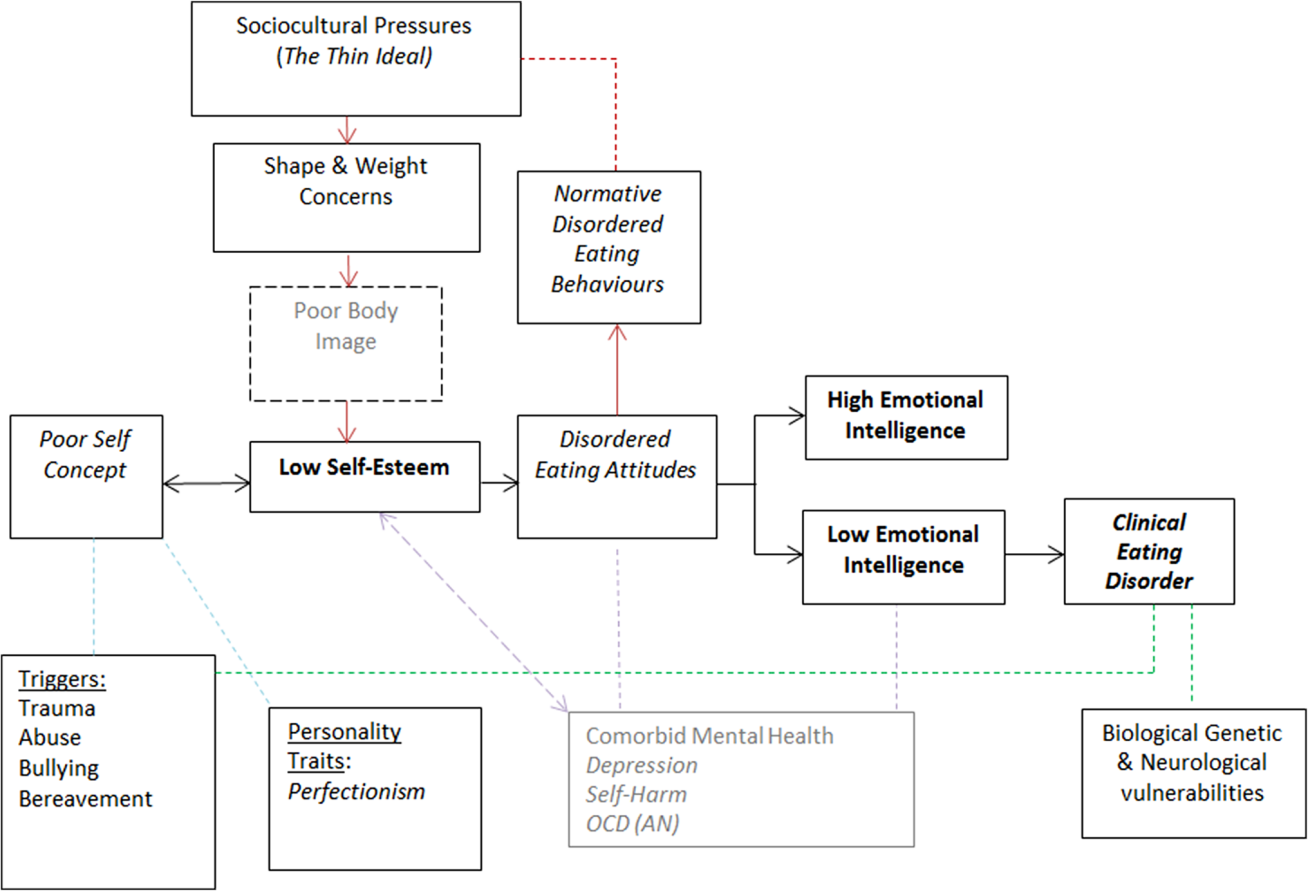
Model exploring the development of disordered eating attitudes and behaviours

This modelling may help to explain why some triggers such as low self-esteem are managed effectively by the majority of the general population with no negative impact; however, a small number will go on to develop an eating disorder. It may be inferred that EI can act as a buffer to moderate against the harmful negative effects of triggering events or low self-esteem, as modelling in Fig. 1. Those with low EI may become vulnerable due to the lack of emotional resources at their disposal, leaving individuals unable to cope, therefore, an alternative is sought [5]. As found in previous qualitative examination of emotional intelligence for eating disordered individuals, it was found that the emotional function that the eating disorder provided acted as a mediator between more normative disordered eating attitudes and the development of more clinical and dangerous symptomology [46]. This supports our understanding of eating disorders as associated with coping and regulation of negative emotion previous explored within the literature [10–12].

Examination of the EI subscales was carried out to provide additional understanding of how EI deficits impact on eating disorder development and maintenance. Appraisal of one’s own emotions, emotional regulation and optimism were found to be significantly lower in all measures of disordered eating, with emotional utilisation significantly lower in relation to attitudes and behaviour scores, suggesting that these elements of EI may be more important in understanding eating disorder aetiology. This is supported by previous research within the field which has suggested that eating disorders occur as a result of emotional dysfunction and regulation difficulties [6–8, 13, 25]. This suggests that disordered eating behaviours may occur due to an individual’s inability to regulate their emotions in response to stressors or triggering events. For example, while low self-esteem is widely considered a core feature that underpins disordered eating psychopathology [60], the co-occurring low abilities to regulate, express and manage emotions have the role to act as a vulnerability factor for individuals with low self-esteem to engage with disordered eating as a form of coping.

## Limitations

It must be acknowledged that the study faced a number of limitations that should be accounted for and addressed within future research. The cross-sectional design of this study limits the conclusions that may be made regarding whether EI deficits contribute to either the onset or the maintenance of eating disorders. As a result, participants included in the analysis are not well distributed or representative in relation to gender, age or history of clinical diagnosis. Future work should consider exploring this relationship in populations that are often underrepresented, e.g. males, as well as replicating this work in clinical samples to further understand the potential unique role EI plays for this group. Furthermore, due to the skewed distribution of EAT-26 scores and population sizes being considerably smaller for the clinical sample, analysis remains simplistic and underpowered to perform mediation analysis. Further research is necessary to apply the theoretical modelling outcome from this study into mediation analysis to further understand the nature of this pathway. A further limitation to address is the use of self-report measurements. All the measurement tools used in the study used self-report scales in which participants reflected on their own perception of their skills, attitudes and behaviours. While each of the tools used has been previously validated, research has shown that the individual’s perception of themselves and their skills are often incompatible to their true abilities.

## Conclusion

The role of EI has been well identified in the literature with these findings showing that lower EI correlates with the development of eating disorders. The results from this study uncovered that some core elements of the EI construct such as appraisal of one’s own emotions and emotional regulation of emotions may be salient to this relationship than other aspects of EI. By understanding the nature of this relationship between EI and eating disorders, and the core aspects of EI that are related to disordered eating, this will support targeted interventions that may support treatments to focus more effectively on the core facets of the disorder rather than more broadly with the vast area of emotions.
